# An Efficient Protocol for Model Legume Root Protoplast Isolation and Transformation

**DOI:** 10.3389/fpls.2018.00670

**Published:** 2018-06-04

**Authors:** Ning Jia, Yali Zhu, Fang Xie

**Affiliations:** ^1^National Key Laboratory of Plant Molecular Genetics, CAS Center for Excellence in Molecular Plant Sciences, Shanghai Institute of Plant Physiology and Ecology, Chinese Academy of Sciences, Shanghai, China; ^2^University of Chinese Academy of Sciences, Beijing, China

**Keywords:** legumes, root protoplasts, PEG-mediated transformation, transient gene expression, symbiosis

## Abstract

Transient gene expression systems using protoplasts have been widely used for rapid functional characterization of genes and high-throughput analysis in many model and crop species. Here, we describe a simplified and highly efficient root protoplast isolation and transient expression system in the model legumes *Lotus japonicus* and *Medicago truncatula.* Firstly, we presented an efficient protocol for isolating protoplasts from *L. japonicus* and *M. truncatula* roots. We then established an efficient transient expression system in these legumes root protoplasts. Using this protocol, the subcellular localization of two symbiosis related proteins (SYMRK and ERN1) were visualized in the plasma membrane and nuclei, respectively. Collectively, this efficient protoplast isolation and transformation protocol is sufficient for studies on protein subcellular localization, and should be suitable for many other molecular biology applications.

## Introduction

Legumes are the third largest group of angiosperms and include many very important food, feed and biofuel crops. Moreover, legumes have evolved a symbiotic interaction with rhizobia bacteria to efficiently fix nitrogen (Masson-Boivin et al., 2009), this nutrient source not only can satisfy its own growth, but also provides nitrogen for other plants grown in rotation or intercropped with legume plants. Nitrogen is one of the most important limiting factors to plant growth and crop yield. Modern agriculture relies heavily on industrial fertilizers to achieve high yields. Industrial nitrogen fixation is a highly energy consuming process. In addition, applying chemical fertilizers is a largely inefficient process, as more than half the fertilizers are lost to leaching, resulting in significant environmental problems, such as eutrophication (Galloway et al., 2008). Therefore, the nitrogen fixation ability of legumes provides them with a distinct advantage over other plant species in agriculture. Detailed investigation of the molecular mechanisms of the symbiosis may help us understand the essence of this phenomenon and provide scientific knowledge for exploring non-legume nitrogen fixation thus reduce the use of industrial fertilizers in future (Perrine-Walker et al., 2007; Rogers and Oldroyd, 2014).

The genome sequence of several model legume species, such as *Medicago truncatula*, *Lotus Japonicus*, and *Glycine max* been published recently (Sato et al., 2008; Schmutz et al., 2010; Young et al., 2011). Several mutant collections have been established by EMS chemical induction (Perry et al., 2009), *LORE1* (Fukai et al., 2012; Urbanski et al., 2012) or *Tnt1* insertion (Tadege et al., 2008) and fast neutron bombardment (FNB) mutagenesis (Chen et al., 2017). Many of the key components involved in symbiosis signal transduction, rhizobial infection and nodule organogenesis have been successively identified in these models (Svistoonoff et al., 2014). However, due to long life cycle of the plants, and the time consuming and low efficiency of the stable genetic transformation systems in these model legumes, the progress of the molecular mechanisms studies of these symbiotic proteins has been greatly limited. Some heterologous systems such as tobacco and Arabidopsis have been used for functional characterization of legume proteins, however, the expression of proteins in heterologous systems has certain limitations. For example, the encoded proteins of some Arabidopsis genes introduced into rice for subcellular localization have been shown to mis-localize (Zhang et al., 2011). In addition, the nodulation process occurs in legume roots and is a very host-specific process. Rhizobia secrete signal molecules, such as Nod factor (Valera and Alexander, 1965), which are very important for rhizobial infection and nodule organogenesis (Oldroyd and Downie, 2008), and heterologous systems cannot respond properly to this signal molecules. The plant protoplast system provides a complementary or alternative stable transgenic system for many types of gene functional studies, such as protein subcellular localization, *in vivo* protein-protein interaction etc. However, an efficient isolation and transient expression system in legumes root protoplast has not yet been established.

Because of the presence of cell walls, many routine molecular biology techniques in animal cells are difficult to perform in plant cells. However, plant protoplasts without cell walls behave similarly to animal cells *in vitro* thus offer a physiological and versatile cell-based experimental system. It is the first report on successful isolation of tomato protoplast in 1960 by Cocking (Cocking, 1960). By using different methods such as PEG-calcium fusion, microinjection, and electroporation, macromolecules including DNA, RNA and proteins can be delivered to protoplasts. By using tobacco mesophyll protoplasts, Fischer and Hain performed the first successful experiment for the transfection into protoplasts (Fischer and Hain, 1995). Subsequently, efficient systems for transient gene expression using protoplasts have been successfully established in different model and crop species including Arabidopsis (Yoo et al., 2007), tobacco (Fischer and Hain, 1995), maize (Sheen, 2001), rice (Zhang et al., 2011), perennial ryegrass (Yu et al., 2017) and *Brachypodium* (Hong et al., 2012). However, efficient systems for transient gene expression mostly focused on leaf mesophyll protoplasts. Root system are important for nutrient intake and response to biotic or abiotic environment. Nitrogen fixation between rhizobia and legume occurs in the host plant roots. However, the root protoplast isolation and transfection protocol in model legumes have not been established. We are trying to isolate legume root protoplasts for functional study of genes related to symbiotic nitrogen fixation and it will be helpful for other studies as well.

In this study, we report a simplified and highly efficient transient gene expression system using root protoplasts from *L. japonicus* and *M. truncatula*. And we also applied this system to get correct subcellular localization of classic genes in nodulation. The application of the new technique in legumes will significantly shorten the time for characterization of gene function.

## Materials and Methods

### Reagents

• Cellulase Onozuka R-10 (Yakult Pharmaceutical Ind. Co., Japan);
• Macerozyme Onozuka R-10 (Yakult Pharmaceutical Ind. Co., Japan);
• D-Sorbitol (Sigma, cat. no. S3889);
• Potassium chloride (KCl; Sigma-Aldrich, cat. no. P3911);
• Calcium chloride dehydrate (CaCl_2_; Sigma, cat. no. C7902);
• Sodium chloride (NaCl; Sigma-Aldrich, cat. no. S9888);
• Magnesium chloride hexa-hydrate (MgCl_2_. 6 H2O; Sigma-Aldrich, cat. no. 9272);
• BSA (Shanghai Bo AO Biotech Co., Ltd., China);
• MES hydrate (Sigma, cat. no. M8250);
• Cell-wall degrading enzyme mix viscozyme (Sigma, cat. no. V2010);
• PEG4000 (Sigma-Aldrich, cat. no. 81240);
• Plasmid Maxi Kit (QIAGEN, cat. no. 12163);
• Gamborg B-5 Basal Medium (Phytotechnology, cat. no. G398);
• T4 DNA Ligase (Takara, cat. no. 2011A);
• Bam HI-HF (NEB, cat. no. R3136S);
• Stu I (NEB, cat. no. R0187S).

### Reagents Setup

• **Digestion solution** (always prepare freshly):
• Prepare 10 mM MES (pH 5.7) containing 1.5% (wt/vol) cellulase R-10, 2% (wt/vol) macerozyme R-10, 0.4M D-sorbitol and 10 mM CaCl_2_. Warm the solution at 55^°^C for 10 min and cool it slowly to room temperature (25^°^C). At the last, add 5% cell-wall degrading enzyme mix viscozyme and 1% BSA.
• **W5 solution:** 154 mM NaCl, 125 mM CaCl_2_ and 5 mM KCl and 10 mM MES (pH 5.7). The prepared W5 solution can be stored in the refrigerator for several weeks.
• **WI solution:** 0.4 M D-sorbitol, 20 mM KCl and 4 mM MES (pH 5.7). The prepared WI solution can be stored at room temperature.
• **DMG solution:** 0.4 M D-sorbitol, 15 mM MgCl_2_ and 4 mM MES (pH 5.7). The prepared DMG solution can be stored at room temperature.
• **PEG–calcium transfection solution:** 20% (wt/vol) PEG4000, 0.2 M D-sorbitol and 100 mM CaCl_2_.

### Equipment

• Fluorescence microscope (Nikon ECLIPSE Ni; Nikon);
• Confocal laser scanning microscope (Leica TCS SP8; Leica);
• Millex-HV filter: Unit-33 mm-0.45 μm (Merck, cat. no. SLHV033RB);
• Cell Strainer, 300 Screen Mesh, Hand Handle (56 μm, Sangon biotech, cat. no. F513442-0001);
• 6-well TC Treated Plates (Sangon biotech, cat. no. F603201-0001);
• Petri dishes 40 × 9 mm (Sangon biotech, cat. no. F611001-0001);
• pH meter (Five Easy pH; Mettler-Toledo);
• Syringe 5 ml (Shanghai Misawa Medical Industry Co., Ltd., China);
• 10-ml round-bottomed Centrifuge tube (Sangon biotech, cat. no. F600889-0001);
• Microscope Cover glassed 22 × 22-1 (Fisherbrand, lot no. 12-542-B);
• Hemacytometer (Shanghai Qiujing Biochemical Reagent Instrument Co., Ltd., China).

## Stepwise Procedures

**FIGURE 1:**
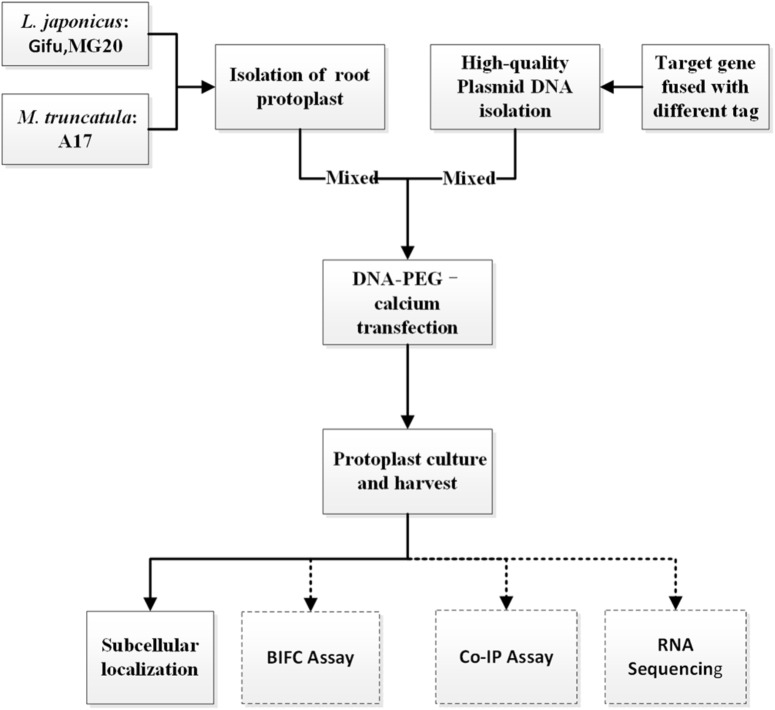
An overview flowchart describing transient gene expression systems using legume root protoplasts.

To establish an efficient transient expression protoplast system for legumes root, we evaluated the protocol established for protoplast isolation from *L. japonicus* MG20 root by modifying various experimental parameters including enzyme digestion time and concentrations, solution recipes, culture medium etc. (**Figure 1**; Den Herder et al., 2012). The entire procedure is divided into six main stages from A to F. (A) Plant growth, (B) Vector construction and plasmid preparation, (C) Isolation of protoplasts from legumes roots, (D) DNA-PEG-calcium transfection, (E) Protoplast culture and harvest, and (F) Images are obtained by laser scanning confocal microscopy.

**CRITICAL:** Except for different plant growth conditions, this protocol (solution recipes and step-by-step) has also applied to Gifu and *M. truncatula* A17.

### Stage A. Plant Growth

**Time**: 6–10 day

*Lotus japonicus* ecotype Gifu and Miyakojima (MG20), *M. truncatula* ecotype A17 were used. The seeds were scarified with sandpaper or immersed for 5–7 min in concentrated H_2_SO_4_ (for *M. truncatula*), then surfaced sterilized with 10% sodium hypochlorite solution for 5–10 min, washed 5 times in sterile H_2_O and kept overnight in water. Imbibed seeds were transferred to 0.8% DWA (Distilled Water Agar) plate and germinated in dark. *M. truncatula* seeds were placed in 4 degree freezers for 3 days before germinated in dark. Then the germinated seedlings were transferred into 1 × Gamborg B5 square plates with a sterilized filter paper placed on the top of the medium as to cover half of the petri dish, germinated seeds are placed on the border of the filter paper so that emerging roots will stay attached to it. The plates were vertical positioned in a controlled growth chamber (For Gifu, 2 days in the dark and 7–8 days in 16 h light/8 h dark; For MG20, 1 day in the dark and 5–6 days in 16 h light/ 8 h dark. For *M. truncatula* A17, seeds were germinated overnight at room temperature, then 5–6 days in 16 h light/8 h dark growth chamber).

**CRITICAL:** The growth status of the plant is very important for root protoplasts isolation and transfection. Healthy plant growing on the 1 × B5 medium instead of 1/2 × B5 can be recommend isolating root protoplasts. Please avoid isolating protoplasts from stressed or older root tissue.

### Stage B. Vector Construction and Plasmid Preparation

**Time**: 2 days

To generate 35S::SYMRK-GFP and 35S::ERN1-GFP construct, the SYMRK coding region (CDS) and ERN1 CDS was PCR-amplified with cDNA from *L. japonicus* seedlings using 5′- ggggggatcc ATGATGGAGTTACCAGCTA-3′ and 5′- ggggaggcctTCTCGGCTGTGGGTGAGAC -3′ for SYMRK, 5′- ggggggatcc ATGGAGATTCAATTCCAGC -3′ and 5′- ggggaggcct ACAGAACAATGAGCACAAGG -3′ for ERN1. The PCR fragment was fused to N termini of GFP in vector pUC19 digested with *Bam* H I /*Stu* I. The constructs were confirmed by sequencing.

For protoplast transfection, high concentration and purity of plasmid DNA is required. Using the QIAGEN MAXI Kit, we were able to isolate sufficiently high-quality plasmid using the manufacturer’s suggested protocols. The extracted plasmid DNA quality was verified using a nanodrop machine and by running a sample on an agarose gel.

**PAUSE POINT:** High concentration and purity of plasmids can be stored at a -20^°^C refrigerator for several months.

**CRITICAL:** We recommend using small size plasmids, because transfection efficiency is higher than large size binary vectors.

**CRITICAL:** High concentration and purity of plasmids DNA are required for protoplasts transfection, and we extract plasmids using the CsCl gradient if necessary.

### Stage C. Isolation of Protoplasts From Legumes Roots

**Time**: 8–10 h

(1) The roots of 6-day-old MG20 seedlings were used for protoplast isolation.
(2) Vigorously growing roots from 6-day-old seedling were used.
(3) Young root tissues from 100 seedlings were cut into 1 mm slices and then dipped into 10 ml of digestion solution. At this step, the amount of digestion solution should be enough to soak all root tissues.
**CRITICAL:** It is difficult to distinguish root protoplasts because color of root tissue is similar to digestion solution. Hypocotyl can be cut in part to show green as an indicator for root protoplasts.
(4) Incubate for 8–12 h in the digestion solution without shaking at 23^°^C in the dark. After digestion, the solution should display a strong brown color, which indicates the release of protoplasts. If the incubation time is too long, the protoplasts will be stressed.
**CRITICAL:** Older roots may need longer digestion time, and there are obvious small bubbles in the tissue indicating that the digestion is complete.
**CRITICAL:** Due to root tissue are denser than leaves, we gently clip root tissues by a large pair of tweezers to push protoplasts out by mechanical force.
(5) Check for the release of root protoplasts in the solution under the microscope; the size of MG20 root protoplasts is approximately 30–50 μm. To remove undigested root tissue, the protoplasts were collected by filtration through 50 μm nylon meshes and diluted with an equal volume of pre-chilled W5 solution. Our method generated approximately 1 × 10^5^ cells varying from 10 to 50 μm in size.
**CRITICAL:** The size of root protoplasts is smaller than leaf, so we filter cells by 300 screen mesh with smaller aperture. To get cleaner root protoplasts, we can filter it twice.
(6) Centrifuge at 400 RCF 4^°^C for 2 min in a swinging-bucket rotor. After centrifugation, discard as much supernatant as possible and re-suspend the protoplasts in the 10 ml pre-chilled W5 solution.
**CRITICAL:** Use the swinging-bucket rotor instead of the corner rotor.
(7) Pellet the protoplasts by centrifugation at 400 RCF 4^°^C for 2 min in a swinging–bucket rotor. Add 5 ml pre-chilled W5 solution and re-suspend the protoplasts in a 10-ml round-bottomed tube.
**CRITICAL:** We suggest using round-bottomed tube in the whole experiments.
(8) Keep protoplasts on ice for 30–60 min. After 15 min protoplasts should begin to settle at the bottom of the tube by gravity. Remove as much of the W5 solution as possible without touching the protoplast pellet. We diluted a suitable concentration of the protoplasts for efficient transfection in DMG solution by using hemacytometer.
**CRITICAL:** The suitable concentration of the protoplasts is very critical for transfection efficiency.

### Stage D. DNA-PEG-Calcium Transfection

**Time**: 30 min

(1) Add 20 μL plasmid DNA (plasmid DNA concentration about 1 μg/μL, size from 5 to 10 kb) to a 5-mL-round-bottom tube.
(2) Mix 200 μL protoplasts (10^4^ protoplasts) and plasmid DNA gently.
(3) Add 220 μL with an equal volume of PEG solution, and then mix well by gently tapping the tube. Incubate for 5 min in the room temperature.
(4) Terminate transfection process by adding 880–900 μL W5 solution at room temperature and mix completely by gently rocking or inverting the tube.
(5) Centrifuge at 400 RCF 4^°^C for 2 min using a swinging-bucket rotor and discard supernatant.
(6) Re-suspend protoplasts gently with 2 mL WI solution and transfer mixture to each well of a 6-well tissue culture plate.
**CRITICAL:** WI solution recipes can only maintain the basic living of root protoplasts.

### Stage E. Protoplast Culture and Harvest

**Time**: 8–20 h

(1) Incubate protoplasts at room temperature (20 ^°^C -25^°^C) for 12-20h in the dark.
**CRITICAL:** Because roots belong to underground tissues, we culture protoplast in the darkness.
(2) Harvest protoplasts by centrifugation at 400 RCF 4^°^C for 2 min a swinging-bucket rotor.
(3) Discard the supernatant and re-suspend protoplasts for further analysis.

### Stage F. Images Are Obtained by Laser Scanning Confocal Microscopy

**Time**: 1 day

Root protoplasts were visualized using SP8 confocal microscope (Leica TCS 8 SP8). The filter sets used for excitation (Ex) and emission (Em) were as follows: GFP, 488 nm (Ex)/from 498 to 550 nm (Em); bright field, 633 nm. Signals were captured in multi-channel mode. All fluorescence experiments were independently repeated at least three times.

## Timing

Stage A: 6–10 days;
Stage B: 2 days;
Stage C: 8–10 h;
Stage D: 30 min;
Stage E: 8–20 h;
Stage F: 1 day;

## Anticipated Results

### Highly Protoplast Isolation Efficiency

**FIGURE 2:**
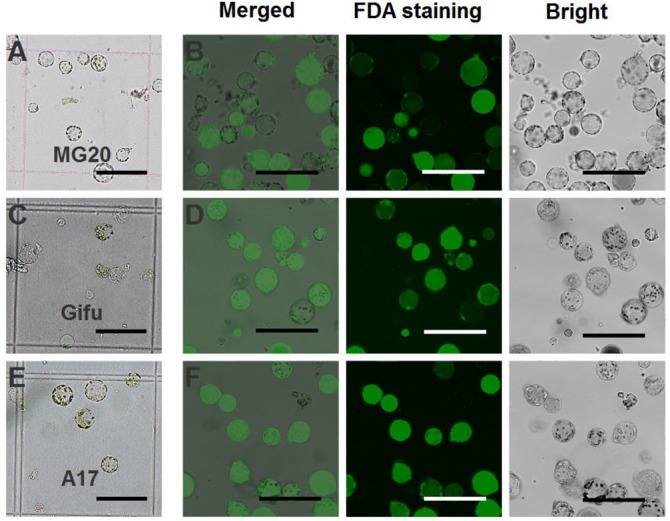
Isolation of protoplasts from legumes roots. Isolated root protoplasts from **(A)** 6-day-old *L. japonicus* MG20 seedling. **(C)** 8-day-old *L. japonicus* Gifu seedling. **(E)** 5-day-old *M. truncatula* A17 seedling. FDA staining of root protoplasts from **(B)** MG20. **(D)** Gifu. **(F)** A17. Image of protoplasts obtained under a Nikon microscope with a 20 × objective. Scale bar = 100 μm.

For *L. japonicus* MG20, our protocol generated approximately 1 × 10^5^ cells varying from 10 to 50 μm in size (**Figure 2A**). Above 90% of isolated root protoplasts were healthy by fluorescein diacetate (FDA) staining (**Figure 2B** and **Table 1**). We diluted the suitable concentration of the protoplasts (1 × 10^4^ cells per transfection) for efficient transfection by using a hemacytometer under a microscope.

**Table 1:** Viability and transfection efficiency of root protoplasts by expression of vector 2 × 35S::GFP-pUC19.

Ecotype	No	Protoplast viability	Transfection efficiency
*L. japonicus* MG20	1	97.9%	61.5%
	2	96.8%	72.7%
	3	99.1%	58.8%
	4	98.1%	68.1%
*M. truncatula* A17	1	98.5%	47.3%
	2	94.9%	53.3%
	3	95.7%	77.7%
			
*L. japonicus* Gifu	1	97.1%	51.1%
	2	93.2%	46.1%
	3	97.9%	62.3%


The root protoplasts from other ecotype (*L. japonicus* Gifu and *M. truncatula* A17) were isolated following the protocol from *L. japonicus* MG20. 8-day-old Gifu and 5-day-old A17 seedlings were prepared for protoplast isolation, but compared to MG20, their yield of root protoplasts is lower (**Figures 2C,E**). FDA staining show that the viability of the cells was no different than root protoplasts from MG20 (**Figures 2D,F**).

### Highly Efficient Transient Transfection

**FIGURE 3:**
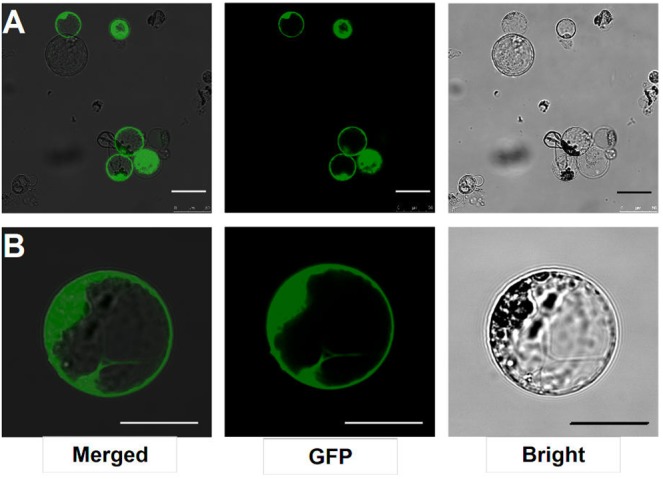
Transient expression GFP-pUC19 in *L. japonicus* MG20 root protoplasts. **(A)** Detection of transfection efficiency by expression of vector 2 × 35S::GFP-pUC19. More than 60% of root protoplasts were transfected successfully with the GFP reporter. **(B)** Detail of expression patterns in the root protoplasts transfection by 2 × 35S::GFP-pUC19 are shown under Confocal microscopy (Leica TCS SP8). Scale bars = 25 μm.

After transfection with the 2 × 35S::GFP plasmid (pUC19-GFP) by using the PEG-mediated transfection approach and incubation for 12 h, the GFP fluorescence was detected both in the cytoplasm and nucleus of the roots protoplasts (**Figure 3B**). In general, root protoplast transfection efficiencies of approximately 60–70% were achieved with this protocol (**Figure 3A** and **Table 1**).

### SYMRK and ERN1 Protein Subcellular Localization in *Lotus* Root Protoplasts

**FIGURE 4:**
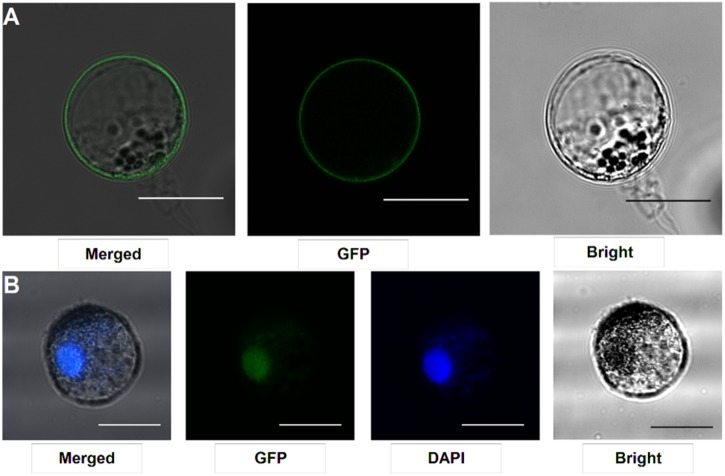
Subcellular localization analysis in *L. japonicus* root protoplasts. **(A)** SYMRK-GFP targeted to plasma membrane in Gifu root protoplasts. **(B)** ERN1-GFP nuclear-localized expression patterns in Gifu root protoplasts, the nucleus-localization was merged with DAPI staining. Scale bars = 25 μm. Transfection efficiency (SYMRK) = 10.7%; Transfection efficiency (ERN1) = 13.6%.

Our protocol can be applied to protein subcellular localization. To test this application, we first cloned two symbiosis related genes (*SYMRK and ERN1*). SYMRK, a symbiosis receptor-like kinase, was previously shown to localize in plasma membrane using hairy root transformation (Stracke et al., 2002; Den Herder et al., 2012). Using the current protocol, we were able to confirm its subcellular localization of plasma membrane in our roots protoplasts system (**Figure 4A**). ERN1, a AP2/ERF type transcription factor required for nodulation, was shown localized in the nucleus in *Lotus* root protoplasts by overlap with DAPI DNA stain (**Figure 4B**) (Andriankaja et al., 2007; Cerri et al., 2017). Its subcellular localization in our homologous protoplast system was consistent with previous reports.

## Author Contributions

NJ contributed to design of this research, performed the experiments, and wrote the manuscript. YZ performed the research. NJ and FX wrote the paper and all the authors approved the manuscript.

## Conflict of Interest Statement

The authors declare that the research was conducted in the absence of any commercial or financial relationships that could be construed as a potential conflict of interest. The reviewer XL and handling Editor declared their shared affiliation.
